# Clinical and radiological outcomes after conservative treatment of TB spondylitis: is the 15 years’ follow-up in the MRC study long enough?

**DOI:** 10.1007/s00586-012-2332-x

**Published:** 2012-05-08

**Authors:** W. Y. Cheung, Keith D. K. Luk

**Affiliations:** Department of Orthopedics and Traumatology, The University of Hong Kong, Pokfulam, Hong Kong SAR, China

**Keywords:** Tuberculosis, Spine, Conservative treatment, Outcomes

## Abstract

**Introduction:**

Tuberculosis of the spine is a still a common disease entity, not only in developing countries but is also returning in developed countries especially in the immune-compromised patients. Conservative treatment with chemotherapy is still the main stay of treatment. This article focuses on the clinical and radiological outcomes, and problems with conservative treatment.

**Method:**

The available literature of anti-tuberculosis chemotherapy in managing spinal tuberculosis was reviewed. Data sources included relevant literature of the English language identified through Medline search from 1946 to 2011. Personal experience and unpublished reviews from the authors’ institution were also included.

**Results:**

Although majority of patients respond well to anti-tuberculosis chemotherapy, about 15 % of them develop paradoxical response. The Medical Research Council (MRC) studies have shown that for patients without significant neurological deficits, operative and conservative treatment could produce the same clinical outcome at 15 years follow-up. Patients treated operatively with debridement and spinal fusion with strut graft had faster bony fusion and less kyphotic deformity. In contrast, those treated with drugs alone or with simple debridement without fusion may result in disease reactivation, severe kyphosis or late instability, which in turn may lead to late-onset Pott’s paraplegia, back pain, sagittal imbalance and compromised pulmonary function that are difficult or risky to treat.

**Conclusion:**

Recognition of the clinical and radiologic features of these late sequels is important for the management. Prevention of deformity in the early disease has been added to the modern standard of treatment of TB spine.

## Introduction

Tuberculosis is still a common infectious disease in the world. Every year, 10 million people are newly infected, with about 95 % of cases being in developing countries. The World Health Organization has estimated that China alone has 1.4 million new cases every year and there are 1.81 million deaths from TB in Asia each year. The USA Center for Disease Control (CDC) has predicted that the number of new diagnoses of active TB worldwide would increase from 7.5 to 11.8 million per year. The incidence of the disease would rise from 143 to 173 per 100,000 and deaths due to TB would climb from 2.5 to 3.5 million or more per year [1, 2]. Such increase in incidence was attributed to increase in life expectancy of people with chronic debilitating diseases, for example chronic renal failure, diabetes mellitus and post-organ transplantation, and also an increase in HIV infection. In some African countries, the number of reported TB cases has doubled or even tripled from 2001 to 2003 because of the spread of HIV/AIDS [1, 2]. While TB commonly infects the lungs, it is located in the spine in 3–5 % of people. Tuberculous spondylitis, although less common, is the most dangerous form of skeletal TB [3, 4].

## Pathophysiology

Tuberculosis of the spine is a potentially life-threatening infection caused by *Mycobacterium tuberculosis*. It is an aerobic, weakly Gram-positive bacillus with a thick cell wall containing mycolic acid, which renders it acid fast. The bacteria commonly reach the spine by hematogenous spread, so the vertebral bodies are usually affected first. They are then taken up by macrophages through phagocytosis and subsequently the delayed hypersensitivity immune response is stimulated, producing cytokines, which lead to recruitment of monocytes, lymphocytes and macrophages. These infected inflammatory cells then form a granuloma and the macrophages differentiate into foam cells, giant cells and epithelioid cells. The center of the granuloma caseates and becomes necrotic. The infection can cause pain and destroy the bone, making the vertebral bodies collapse, leading to kyphosis. Tuberculosis abscesses expand, following the path of least resistance, and contain necrotic debris. The infection spreads beneath the anterior and posterior longitudinal ligaments to the adjacent levels. Skin sinuses may form and drain spontaneously. The infection may also result in neurological damage. Sometimes, nerve roots may be compressed causing pain along the roots or radiculopathy, but more commonly spinal cord or cauda equina compression may lead to myelopathy or paraplegia. It may happen in early active disease due to spinal cord compression by inflammatory tissues, epidural abscess, protruded intervertebral disc, pachymeningitis or spinal subluxation. It may also happen years after the initial TB infection, known as late-onset paraplegia, which is commonly due to severe kyphosis from non-union with chronic spinal cord compression or spinal cord atrophy, with or without reactivation of the infection. Apart from spinal cord compression at the kyphus, patients may develop late-onset paraplegia due to spinal stenosis above the healed kyphosis [5]. In order to compensate for the kyphotic deformity in the thoracolumbar junction as a result of the TB infection, the unaffected thoracic and lumbar spine cranially and caudally go into compensatory hyperlordosis to achieve overall sagittal balance. Such hyper-extension of the adjacent levels can lead to early facet joints degeneration, spinal stenosis and neurological deficits.

## Clinical presentation and diagnosis

Slowly progressive constitutional symptoms are predominant in the early stages of the disease, including generalized weakness, malaise, night sweats, fever and weight loss. Pain is a late symptom when bone collapse occurs. The patients may also present with neurological deficits with lower limb weakness and numbness known as Pott’s paraplegia as a result of spinal cord or cauda equina compression. In severe disease, the patients may even lose their urinary and bowel control. Since the development of compression is slow and progressive, neurological signs usually occur late. Jain et al. [6] calculated that the spinal canal can accommodate 76 % encroachment on CT scan without neurological abnormality. Rarely, cervical involvement can cause hoarseness because of recurrent laryngeal nerve paralysis, dysphagia and respiratory stridor (known as Millar asthma). These symptoms may result from anterior abscess formation in the neck. Sudden death has also been reported with cervical disease after erosion into the great vessels.

Laboratory studies suggest chronic infection with findings including anemia, hypoproteinemia, elevation of erythrocyte sedimentation rate and C-reactive protein. Tuberculin skin testing may be helpful, but is not diagnostic, especially in TB, endemic areas where the population could have had subclinical exposures or received BCG vaccination. It is also not sensitive in immune-compromised patients. Sensitivity of only 56 % with tuberculin testing in rheumatoid arthritis patients on immuno-suppressant with TB infection was reported [7]. Interferon gamma-release assays (IGRA) is a relatively new method for detecting T cells specific for *Mycobacterium tuberculosis* antigens. It has sensitivity of about 83–90 % and is more specific than the tuberculin skin test. Its sensitivity remains high in immune-compromised patients and is not confounded by BCG vaccination. The main limitation of the test is it cannot differentiate active from latent and treated infections [8].

Early radiographic findings include a subtle decrease in one or more disc spaces and localized osteopenia. The paraspinal abscess presents typically as a fusiform radio-opaque shadow against a radiolucent background of the lungs. Later findings include vertebral collapse described by Seddon as “concertina collapse” because of its resemblance to an accordion. Soft tissue swelling and its late calcification are highly predictable radiographic findings. Computed tomography scanning, with contrast, allows better evaluation of the bony destruction and possible instability. Magnetic resonance imaging permits further delineation of the soft tissue components, activity of the disease and status of the spinal cord. Gupta et al. [9] noted that abscess formation and the presence of bone fragments were MRI findings that helped distinguish spinal TB from neoplasia. However, none of these tests is confirmatory for TB.

Definitive diagnosis depends on culture of the organism and requires biopsy of the lesion. Percutaneous biopsies under radiographic or CT control are usually adequate. Camillo et al. [10] reported 29 patients with suspected spinal TB. Epithelioid granulomas were seen in 89 % positive acid-fast bacilli cultures in 83 % and positive acid-fast bacilli smears in 52 %. Percutaneous thoracoscopic or laparoscopic biopsy is another reported option as noted by Dusmet et al. Open biopsy may be necessary if needle biopsy is unsuccessful or if it could be done during the definitive open procedures.

*Mycobacterium tuberculosis* is difficult to culture due to its fastidious growth requirements and slow growth rate. Hence, there is a need for the development of various laboratory methods to allow more effective and earlier diagnosis. Polymerase chain reaction is a commonly employed, non-culture, molecular diagnostic test, which amplifies the DNA of the *Mycobacterium tuberculosis* for identification. The amplified segment of the TB DNA is subsequently detected with the Southern blot hybridization technique. Polymerase chain reaction is highly sensitive (95–98 %) for diagnosing TB from smear-positive and culture-positive cases, but it has lower sensitivity (57–78 %) for smear-negative and culture-positive cases [11].

## Conservative treatment and outcomes

Chemotherapy is the main state of treatment for TB infection. With the advent of specific anti-TB chemotherapy, the clinical course of TB has been changed such that patients rarely die from the disease nowadays. The period of infectivity has also been considerably reduced, relapses avoided and chronicity reduced. There are five first-line anti-tuberculous drugs, namely isoniazid, rifampicin, pyrazinamide, streptomycin and ethambutol. Various treatment regimes have been described and generally 6–12 months of chemotherapy is required [2, 12]. With the increasing incidence of drug-resistant TB worldwide, it is very important to know bacterial sensitivities before commencing chemotherapy. By culture of aspirate or tissue specimens, the sensitivity tests of the cultured tubercle bacilli against each drug can be ascertained.

**Fig. 1:**
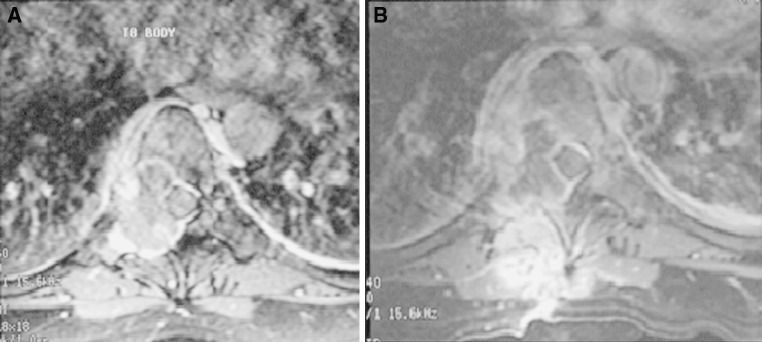
Paradoxical response after anti-TB treatment. **a** Contrast MRI at presentation, **b** contrast MRI after four weeks of anti-TB medications showed increased inflammation, soft tissue swelling and abscesses

**Fig. 2:**
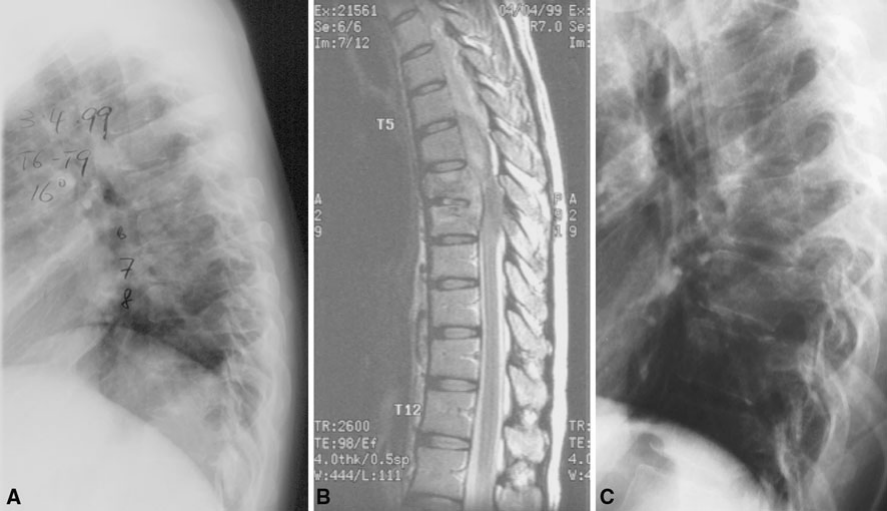
Patient was successfully treated with anti-TB medications. **a**, **b** X-ray and MRI at presentation. **c** X-ray 1 year after treatment showed spontaneous fusion

Though the majority of the patients respond well to anti-TB treatment, paradoxical response occurs in about 15 % of cases [13]. It usually develops 2 weeks to a few months after starting the anti-TB treatment. It is defined as clinical or radiological worsening of pre-existing tuberculous lesions or the development of new lesions not attributable to the normal course of disease, in a patient who initially improved with anti-TB drugs. It is a diagnosis by exclusion, which can only be made after excluding secondary bacterial infection, non-compliance to drug treatment and development of drug resistance. Up to 10 % of patients with central nervous system TB report paradoxical response and this number may be as high as 30 % in HIV-infected patients [14, 15]. The paradoxical response is a component of immune reconstitution inflammatory syndrome or immune restoration syndrome, which results from an exuberant inflammatory response toward incubating opportunistic pathogens [16]. Various reports in the literature have documented an increased incidence and severity of the paradoxical response in HIV-infected patients on highly active antiretroviral therapy (HAART) [15]. Patients demonstrating a paradoxical response are more likely to have lower baseline lymphocyte counts, followed by a surge [13]. This surge may be profound in HIV-infected patients recently started on HAART. Pregnancy may also be a risk factor for the paradoxical response. An example is presented in Figs. 1 and 2. The patient was a pregnant lady at 5 months of gestation when TB of the thoracic spine was diagnosed. She was treated with anti-TB medications, but her symptoms deteriorated 4 weeks after commencing the treatment. Magnetic resonance imaging of the thoracic spine was repeated which showed increased inflammation, soft tissue swelling and abscess formation suggesting progression of the infection or paradoxical response with liquefaction of the caseous material (Fig. 1). Sensitivity test confirmed that the bacteria were susceptible to the medications and drug compliance was ensured. Paradoxical response was diagnosed and anti-TB medications were continued. The infection was successfully treated with spontaneous fusion (Fig. 2).

For patients suffering from active tuberculous infection of the spine with progressive neurological deficit from spinal cord or cauda equina compression, surgical decompression and fusion is indicated. However, the role of surgery for patients without neurological compromise is controversial. To study the outcomes of the conservative and surgical approach to the treatment of TB spine in this group of patients, the Medical Research Council of the UK coordinated a series of important clinical trials. These studies were carried out from 1965 in multiple centers in the world where the disease was prevalent. The patients were carefully selected, documented and followed-up for as long as 15 years prospectively and the results of these works have been published in a series of reports [17–21]. In Rhodesia, Korea and Hong Kong, patients were randomly allocated to the drug treatment group, debridement group or radical debridement plus anterior spinal fusion group. The inclusion criterion was clinical or radiological evidence of TB of any level except the cervical spine. Patients with significant neurological deficit, those who had been given anti-TB drugs for more than 1 year, those who had significant extra-spinal disease and, for the fusion group, those who had more than three levels of vertebral destruction were excluded from the study. All patients were given anti-TB chemotherapy. In the debridement group, the abscess, sequestrum and loose disc fragments were removed to achieve spinal cord decompression and no fusion was performed. In Hong Kong, the radical surgery group received a radical debridement of the necrotic tissue until ‘healthy bleeding bone’ was reached. This was then followed by an anterior strut graft fusion using autologous rib, iliac or fibula grafts. The results reported at 5, 10 and 15 years indicated that all three groups achieved the same 87 % favorable outcome (Table 1). The favorable outcome was defined as no evidence of central nervous system involvement, no sinus or clinically evident abscess, no radiological evidence of disease activity and no restriction of normal physical activity. However, it was also noted that patients treated conservatively had significantly lower fusion rate, 46 % at 5 years and 72 % at 15 years, compared with 85 and 94 %, respectively, in patients who received surgical debridement and fusion (Table 2). The conservatively treated group also had more progression of kyphosis, 21° at 5 years, which further increased to 25° at 15 years. In contrast, the radical debridement and fusion group showed an improvement of 3° at 5 years, and this was maintained at the final 15-year follow-up assessment (Table 3). It is also worth noting that in 5 % of the conservative group, there was an alarming increase of kyphosis from 51 to 70°. So when these data were analyzed in greater detail, it was evident that the radical surgery group treated in Hong Kong did have distinct advantages over the other two groups. There was less kyphotic deformity, much quicker relief of pain, earlier resolution of the sinus tracts and abscesses, and there was no neurologic involvement during treatment in this group.

**Table 1:** Favorable outcome was achieved in 87 % of patients treated with chemotherapy alone, chemotherapy with debridement and chemotherapy with radical debridement and fusion

	No. of patients	Favorable (%)	Doubtful (%)	Unfavorable (%)
Korea chemotherapy alone	271	236 (87)	1 (<1)	34 (13)
Hong Kong chemotherapy and simple debridement	54	47 (87)	0 (0)	7 (13)
Hong Kong chemotherapy, radical debridement and fusion	52	45 (87)	0 (0)	7 (13)

Modified from the 13th report of the Medical Research Council working party on TB of the spine [20]

**Table 2:** Percentages of patients who achieved bony fusion at 18 months, and 5, 10 and 15 years after treatment

	18 months (%)	5 years (%)	10 years (%)	15 years (%)
Korea chemotherapy alone	15	46	73	72
Hong Kong debridement alone	52	84	90	94
Hong Kong debridement and fusion	85	92	97	94

Modified from the 13th report of the Medical Research Council working party on TB of the spine [20]

**Table 3:** Increase in kyphosis in 5 and 15 years after treatment

	Increase in kyphosis in 5 years (°)	Increase in kyphosis in 15 years (°)
Chemotherapy alone	21	25
Chemotherapy and debridement	8	11
Chemotherapy, debridement and fusion	−3	−3

Modified from the 13th report of the Medical Research Council working party on TB of the spine [20]

## Problems of post-tuberculosis kyphosis

**Fig. 3:**
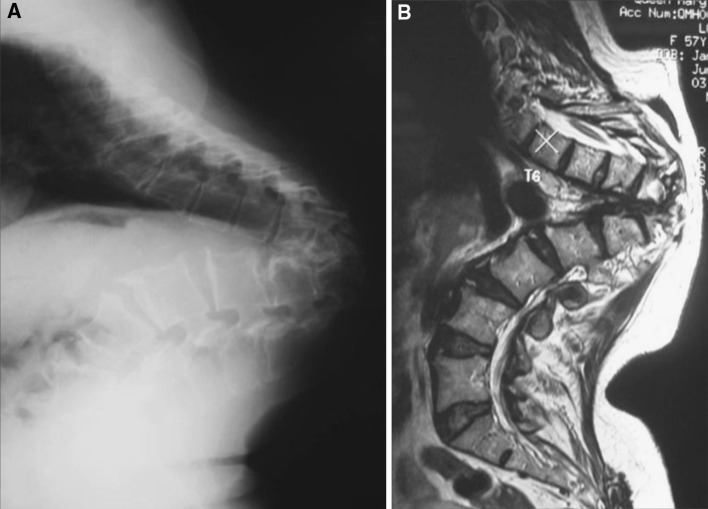
Late-onset Pott’s paraplegia with healed disease. **a** Severe kyphosis at the lower thoracic region. **b** MRI showed no evidence of reactivation of infection

**Fig. 4:**
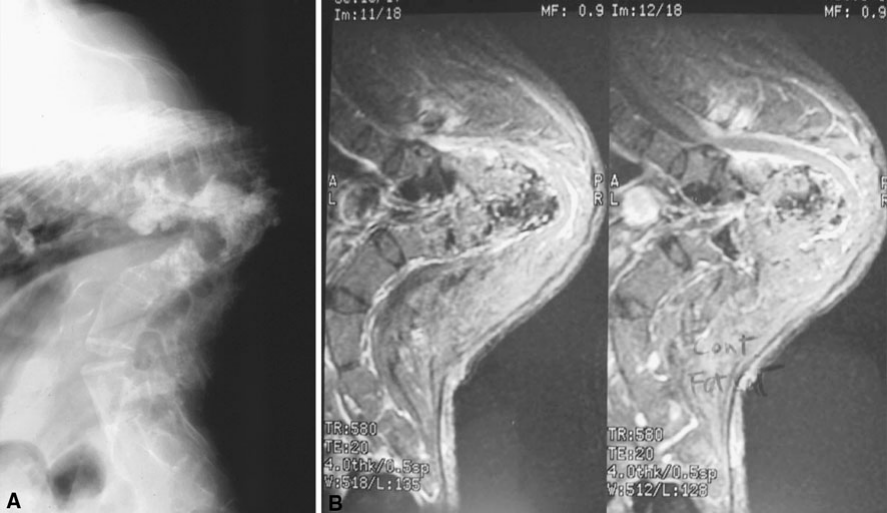
Late-onset Pott’s paraplegia with active disease. **a** Severe kyphosis over the thoracolumbar region with increased bone destruction. **b** MRI showed contrast enhancement at the kyphus

Progressive bony destruction with collapse of the vertebrae leading to severe kyphosis may occur even with successful medical treatment, especially in patients with multiple level disease. In some cases, the kyphosis only stops when the two limbs approximate each other, leading to severe deformity and internal kyphus compressing on the spinal cord. Gross kyphotic deformities after TB infection can pose many problems to the patients. Severe kyphosis in the thoracic and thoracolumbar region can result in significant cardio-respiratory embarrassment. Deformities in the lumbar region can cause severe postural imbalance and shortening of the trunk, leading to increased back pain from muscle fatigue and impingement of the rib cage over the iliac crests. It can also result in body image, self-esteem and other psychological problems. More importantly, prolonged kinking of the spinal cord or cauda equina over the kyphus may lead to late neurological complication known as late-onset Pott’s paraplegia in healed disease (Fig. 3). It is attributed to continuous stretching of the spinal cord over the kyphus. The spinal cord as a result develops inflammatory edema, cord atrophy (gliosis), myelomalacia, and syringomyelia. The long-standing gradual insult to the cord leads to exhaustion of physiologic reserve; hence the late-onset paraplegia. Non-union and instability at the kyphus may also contribute to the late-onset paraplegia. When there is non-union at the kyphus, it opens and closes with movements, resulting in dynamic damage to spinal cord and neurological deficits. Because of the post-TB kyphosis that commonly occurs at the thoracolumbar junction, the compensatory hyperlordosis at the adjacent levels may also lead to accelerated facet joint degeneration, spinal canal stenosis and neurological deficit [5]. In addition to the prolonged kinking of the spinal cord as a result of severe kyphosis, some patients may have reactivation of the infection many years after the initial TB infection, which may lead to deterioration of neurological deficits. It is known as late-onset Pott’s paraplegia with active disease. Besides paraplegia, patients also have evidence of disease reactivation, including pain and tenderness over the kyphus, malaise, weight loss and raised infective parameter. X-ray may show increased bone destruction with bag of calcified caseation and blurring of bone margins, and MRI may show disease activity with high T2 signal and contrast enhancement (Fig. 4).

According to an unpublished retrospective review of 103 patients who suffered from TB of the spine that required surgical interventions in our institution, 41 patients were operated for early active disease and 62 for late complications of the infection. Among the 62 patients in the latter group, 36 were operated for back pain and sagittal imbalance and 26 for late-onset Pott’s paraplegia with 15 (58 %) of them presenting more than 20 years after the initial spinal infection [22]. Similarly, Moon et al. [23] reported about their 33-patient case series with late-onset Pott’s paraplegia from severe post-TB kyphosis. The patients on average presented 16 years, ranging from 4 to 27 years, after the initial infection. So the 15-year follow-up studies comparing conservative and operative treatment for TB spondylitis by the Medical Research Council is insufficient to detect the difference in long-term outcomes between these two groups.

## Poor prognostic factors to develop severe kyphosis

Knowing that severe kyphosis can lead to late complications which are very difficult to treat and radical debridement and fusion can effectively stop progression of kyphosis, a question therefore arises. Can we predict which deformity will progress to severe kyphosis such that early surgical intervention can be done to prevent this? Rajasekaran et al. [24, 25] tried to answer this question by identifying the risk factors in patients who developed severe angular kyphosis. They retrospectively reviewed 90 adult patients who suffered from TB of the spine and found that the amount of vertebral body loss at the start of treatment had a good correlation with the severity of the deformity at the 5-year follow-up. They reported that the deformity at 5 years could be predicted with a fair level of accuracy by the formula *Y* = *a* + *bX*, where *Y* is the kyphotic deformity at the 5-year follow-up, *X* is the pretreatment vertebral body loss, and *a* and *b* are constant values of 5.5 and 30.5. There was an average kyphus angle of 30–35° for the complete destruction of each vertebral body in the dorsal and dorsolumbar region and approximately 20° for the complete loss of each vertebral body in the lumbar region. They recommended surgery for patients with loss of 0.75 of thoracic or thoracolumbar vertebra, or loss of one lumbar vertebra aiming at a final kyphosis of no more than 30°. In another study, they also identified some risk factors for severe kyphosis in the pediatric patients, namely separation of facet joint, posterior retropulsion, lateral translation and toppling over. Patients with more than two of the signs had progression of kyphosis of more than 30° and a final kyphosis of more than 60°. Surgical treatment is therefore also recommended for this group of patients.

## Treatment for severe post-tuberculosis kyphosis

While early surgical intervention for prevention of deformity is relatively simple and usually produces good results, treating severe late symptomatic post-TB kyphosis is difficult and carries high risk. In the past, single-stage correction of deformity had an unacceptable rate of neurologic complications and the amount of correction obtained could be negligible and dissatisfying to the surgeon and the patient. There was also a constant danger of paraplegia because of the need for meticulous debridement of the tissues all around the spinal cord before osteotomy. The presence of side-slip deformity at the apex of the kyphosis makes the procedure even more dangerous, because of the difficulty in locating the spinal canal. To minimize the complications, a sequential procedure was advocated by Yau et al. [26] which involved multiple stages of fitting of the halo-pelvic distraction apparatus, anterior spinal osteotomy and decompression of the spinal cord, slow and gradual spinal distraction, posterior osteotomy and fusion, additional spinal distraction, and anterior spine fusion after achieving maximum correction. Even with this technique of staged procedures, there was a 10 % mortality rate and the average amount of correction obtained was only 28 %. Therefore, this surgery was done only in patients in whom the deformity was severe, the disease was still active and paraplegia or death from chest complications was imminent.

**Fig. 5:**
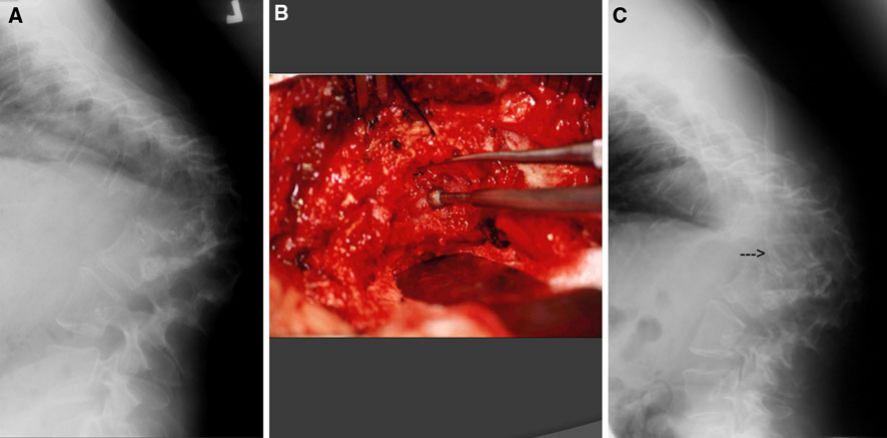
**a** Severe post-tubercular kyphosis, **b** internal kyphectomy for spinal cord (*arrow*) decompression, and **c** post-operative x-ray showed internal kyphectomy and bone graft in position (*arrow*)

Although recent advances in spinal instrumentation and intra-operative spinal cord monitoring techniques have made post-TB kyphosis correction surgery safer, it is still a risky surgical undertaking with high chance of neurological complication. Moon et al. [23] reported seven cases of late-onset Pott’s paraplegia with healed disease treated with decompression and spinal fusion; only one patient had neurological improvement after surgery. The severity of paralysis remained unchanged in four patients and deteriorated in two patients after surgery. Hsu et al. [27] reported that 80 % of patients with active disease and 50 % of patients with healed disease had neurological improvement with internal kyphectomy and fusion with strut graft. However, 10 % of patients had neurological deterioration after the surgery. More recently, Rajasekaran et al. [28] described a single-stage closing–opening wedge osteotomy to correct severe kyphosis as a result of TB infection. The procedure is done through a single posterior approach and wide laminectomies, and a wedge of the vertebral column is excised. The anterior column is reconstructed with a cage and the deformity correction is done with posterior closing and anterior opening, fulcruming at the cage. The merits of this procedure are that the spinal column is not excessively shortened or lengthened during the procedure, so the chance of neurological complication should be lower. He reported 17 cases with average kyphosis correction of 57 %, with one patient (6 %) having neurological deterioration after the procedure. Furthermore, one should be careful about the flexibility of the compensatory hyperlordosis proximal and distal to the kyphus before proceeding with this surgical procedure. Particularly if the thoracic hyperlordosis is not reversible, any overcorrection of the thoracolumbar kyphosis may result in stresses being exerted into the cervical segment. Thus, the best indication for this procedure would be for children or young adults where the compensatory curves are still flexible and can be reversed with the segmental instrumentation. For severe kyphosis with paraplegia of healed disease presenting at late adulthood, salvage of slowly progressive neurology is the main goal of treatment. Correction of the rigid and severe spinal deformity is no longer a priority. In our center, we prefer to decompress the internal gibbus and stabilize the kyphosis with strut bone grafting via the costotransversectomy approach for this group of patients [27, 29]. A curved longitudinal incision 6–8 cm lateral to the midline is created, centered over the kyphus. The spine is approached lateral to the erector spinae muscles directly toward the tips of the transverse processes. The adjacent 5–6 cm ribs are then exposed subperiosteally. Two to three transverse processes and the corresponding posterior end of the ribs including the rib heads are excised. The segmental intercostal nerves are identified and held with slings. Great care is taken to remain extrapleural and extraperitoneal. Soft tissue, pleura and peritoneum are mobilized from the pedicles and collapsed vertebral bodies using blunt dissection. The spinal canal is entered by tracing the intercostal nerves and removing the surrounding bone. Crowded pedicles at the apex are removed and the dura is exposed posterior to the posterior longitudinal ligament. Excision of the internal kyphus is performed to decompress the spinal cord and anterior strut fusion is performed by placing multiple rib grafts as far anterior as possible (Fig. 5). Recently, we published our 5-year results for patients with late-onset Pott’s paraplegia of healed disease and severe kyphosis treated with this method [29]. Forty per cent of patients had neurological improvement and none had neurological deterioration after the surgery. Solid bone fusion was demonstrated in all patients at 5 years after surgery. This surgical procedure is technically less demanding; however, it does not correct the kyphosis and sagittal malalignment. It is particularly indicated for older patients with fixed compensatory lordosis or patients with multiple co-morbidities and high surgical risks.

## Conclusion

Tuberculosis of the spine is a serious clinical condition which can lead to pain, kyphosis deformity and neurological complications. For patients without significant neurological deficits, conservative treatment can be expected to give good clinical outcome in 15 years. Though the majority of the patients respond well to medical treatment, paradoxical response occurs in about 15 % of them. Progressive bone destruction with severe kyphosis may still develop in patients with successful medical treatment, especially for those with poor prognostic factors, leading to late complications including late-onset Pott’s paraplegia with active or healed diseases. These late complications commonly present more than 15 years after the initial spinal infection [22, 23], suggesting that the 15-year follow-up study by the Medical Research Council is insufficient to judge the superiority of surgical versus conservative treatment. Since early operative treatment with good debridement and strut fusion can produce higher bone fusion rate and correct kyphotic deformity [17–21], treatment for late-onset complications are difficult and carries high risk [23, 26–29]; prevention of severe spinal deformity at the early stage should be the modern standard of treatment especially for high-risk patients.

## References

[1] Moon MS (1997). Tuberculosis of the spine; controversies and new challenges. Spine.

[2] Moon MS (2007). Tuberculosis of the spine—contemporary thoughts on current issues and perspective views. Curr Orthop.

[3] Jutte PC, Van Loenhout-Rooyackers JH (2006) Routine surgery in addition to chemotherapy for treating spinal tuberculosis. Cochrane Database Syst Rev, Issue 1, Art. No.: CD004532. doi: 10.1002/14651858.CD004532.pub2 10.1002/14651858.CD004532.pub2 PMC6532687 16437489

[4] Watts HG, Lifeso RM (1996). Tuberculosis of bone and joints. J Bone Joint Surg.

[5] Luk KD, Krishna M (1996). Spinal stenosis above a healed tuberculosis kyphosis. A case report. Spine.

[6] Jain AK, Aggarwal A, Mehrotra G (1999). Correlation of canal encroachment with neurological deficit in tuberculosis of spine. Int Orthop (SICOT).

[7] Marques CDL, Duarte ÂLBP, de Lorena VMB, Souza JR, Souza WV, de Miranda Gomes Y, de Carvalho EMF (2009). Evaluation of an interferon gamma assay in the diagnosis of latent tuberculosis infection in patients with rheumatoid arthritis. Rheumatol Int.

[8] Herrera Victor, Perry Sharon, Parsonnet Julie, Banaei Niaz (2011). Clinical application and limitations of interferon-*g* release assays for the diagnosis of latent tuberculosis infection. Clin Pract.

[9] Gupta RK, Agarwal P, Rastogi H, Kumar S, Phadke RV, Krishnani N (1996). Problems in distinguishing spinal tuberculosis from neoplasia on MRI. Neuroradiology.

[10] Camillo FX, Canale ST (2008). Infection of the Spine. Campbell’s Operative Orthopaedics.

[11] Rattan A (2000). PCR for diagnosis of tuberculosis: where are we now?. Indian J Tuberc.

[12] Medical Research Council Working Party on Tuberculosis of the Spine (1993). Controlled trial of short-course regimens of chemotherapy in ambulatory treatment of spinal tuberculosis. J Bone Joint Surg.

[13] Cheng VCC, Yam WC, Woo PCY, Lau SKP, Hung IFN, Wong SPY, Cheung WC, Yuen KY (2003). Risk factors for development of paradoxical response during antituberculosis therapy in HIV-negative patients. Eur J Clin Microbiol Infect Dis.

[14] Gupta M, Bajaj BK, Khawaja G (2003). Paradoxical response in patients with CNS tuberculosis. J Assoc Physicians India.

[15] Breen RA, Smith CJ, Bettinson H (2004). Paradoxical reactions during tuberculosis treatment in patients with and without HIV co-infection. Thorax.

[16] Shelbume SA, Hamill RJ (2003). The immune reconstitution inflammatory syndrome. AIDS Rev.

[17] Medical Research Council Working Party on Tuberculosis of the Spine (1978). Five-year assessments of controlled trials of ambulatory treatment, debridement and anterior spinal fusion in the management of tuberculosis of the spine: studies in Bulawayo (Rhodesia) and in Hong Kong. J Bone Joint Surg Br.

[18] Medical Research Council Working Party on Tuberculosis of the Spine (1982). A ten-year assessment of a controlled trial comparing debridement and anterior spinal fusion in the management of tuberculosis of the spine in patients on standard chemotherapy in Hong Kong. J Bone Joint Surg Br.

[19] Medical Research Council Working Party on Tuberculosis of the Spine (1985). A ten-year assessment of controlled trials of inpatient and outpatient treatment and of plaster-of-paris jacket for tuberculosis of the spine in children on standard chemotherapy. J Bone Joint Surg Br.

[20] Medical Research Council Working Party on Tuberculosis of the Spine (1998). A 15-year assessment of controlled trials of the management of tuberculosis of the spine in Korea and Hong Kong. J Bone Joint Surg Br.

[21] Medical Research Council Working Party on Tuberculosis of the Spine (1974). A controlled trial of anterior spinal fusion and debridement in the surgical management of tuberculosis of the spine in patients on standard chemotherapy: a study in Hong Kong. Br J Surg.

[22] Luk KDK (1999). Tuberculosis of the spine in the new millennium. Eur Spine J.

[23] Moon MS, Moon JL, Moon YW, Kim SS, Kim SS, Sun DH, Choi WT (2003). Pott’s paraplegia in patients with severely deformed dorsal or dorsolumbar spines: treatment and prognosis. Spinal Cord.

[24] Rajasekaran S, Shanmugasundaram TK (1987). Prediction of the angle of gibbus deformity in tuberculosis of the spine. J Bone Joint Surg Am.

[25] Rajasekaran S (2001). The natural history of post-tubercular kyphosis in children. Radiological signs which predict late increase in deformity. J Bone Joint Surg Br.

[26] Yau ACMC, Hsu LCS, O’Brien JP, Hodgson AR (1974). Tuberculosis kyphosis-correction with spinal osteotomy, halo-pelvic distraction and anterior and posterior fusion. J Bone Joint Surg.

[27] Hsu LCS, Cheng CL, Leong JCY (1988). Pott’s paraplegia of late-onset: the cause of compression and results after anterior decompression. J Bone Joint Surg Br.

[28] Rajasekaran S, Vijay K, Shetty AP (2010). Single-stage closing-opening wedge osteotomy of spine to correct severe post-tubercular kyphotic deformities of the spine: a 3-year follow-up of 17 patients. Eur Spine J.

[29] Wong YW, Leong JCY, Luk KDK (2007). Direct internal kyphectomy for severe angular tuberculosis kyphosis. Clin Orthop Relat Res.

